# Activation of Kupffer cells in NAFLD and NASH: mechanisms and therapeutic interventions

**DOI:** 10.3389/fcell.2023.1199519

**Published:** 2023-05-16

**Authors:** Gao-Xin Xu, Song Wei, Chao Yu, Si-Qi Zhao, Wei-Jun Yang, Yong-Heng Feng, Chao Pan, Kun-Xing Yang, Yong Ma

**Affiliations:** Department of General Surgery, Nanjing First Hospital, Nanjing Medical University, Nanjing, China

**Keywords:** NAFLD, NASH, Kupffer cell, activation, treatment, signaling pathway, molecular mechanisms

## Abstract

Non-alcoholic fatty liver disease (NAFLD) and non-alcoholic steatohepatitis (NASH) are emerging as the leading causes of liver disease worldwide. These conditions can lead to cirrhosis, liver cancer, liver failure, and other related ailments. At present, liver transplantation remains the sole treatment option for end-stage NASH, leading to a rapidly growing socioeconomic burden. Kupffer cells (KCs) are a dominant population of macrophages that reside in the liver, playing a crucial role in innate immunity. Their primary function includes phagocytosing exogenous substances, presenting antigens, and triggering immune responses. Moreover, they interact with other liver cells during the pathogenesis of NAFLD, and this crosstalk may either delay or exacerbate disease progression. Stimulation by endogenous signals triggers the activation of KCs, resulting in the expression of various inflammatory factors and chemokines, such as NLRP3, TNF-α, IL-1B, and IL-6, and contributing to the inflammatory cascade. In the past 5 years, significant advances have been made in understanding the biological properties and immune functions of KCs in NAFLD, including their interactions with tissue molecules, underlying molecular mechanisms, signaling pathways, and relevant therapeutic interventions. Having a comprehensive understanding of these mechanisms and characteristics can have enormous potential in guiding future strategies for the prevention and treatment of NAFLD.

## 1 Introduction

Due to the increasing obesity epidemic, non-alcoholic fatty liver disease (NAFLD) has emerged as a major global health concern and is now one of the leading causes of chronic liver disease worldwide (Younossi et al., 2018). NAFLD encompasses non-alcoholic steatohepatitis (NASH), which is diagnosed by histology and is characterized by several interrelated pathological processes, such as hepatic steatosis, inflammatory infiltrates, hepatocyte necrosis, extracellular matrix deposition, and fibrotic response. Therefore, NASH is considered a progressive form of NAFLD (Schuster et al., 2018).

In 2020, a team of international experts introduced the concept of metabolic dysfunction-associated fatty liver disease (MAFLD) to highlight the significance of various metabolic factors in the disease and distinguish it from other chronic liver disorders, such as those induced by alcohol. The panel also recommended specific diagnostic methods that involve assessing various values, such as body mass index (BMI), blood pressure, the presence or absence of type 2 diabetes, triglyceride levels, insulin resistance score, and C-reactive protein level. This shift from an exclusion-based to an inclusion-based diagnosis, together with the proposed diagnostic criteria, will enable healthcare providers to more accurately evaluate the severity of the disease (Fouad et al., 2021); (Eslam et al., 2020).

Available statistics show that the global incidence of NAFLD has been steadily increasing over time, from 25.5% before 2005 to 37.8% after 2016. The incidence varies across different regions, likely due to lifestyle and dietary habits, ethnic disparities, and climatic variations. For example, Japan reports a prevalence of 22.3%, while the United States records a rate of 47.8% (Riazi et al., 2022). Furthermore, recent research has linked the occurrence of NAFLD to the circadian rhythm (Saran et al., 2020).

Although isolated hepatic steatosis may not pose a significant risk, the progression of NAFLD to NASH significantly increases the chances of developing cirrhosis, liver failure, or even hepatocellular carcinoma (Friedman et al., 2018). Additionally, NAFLD is linked with various extrahepatic complications, including but not limited to heart disease, stroke, chronic kidney disease, type 2 diabetes, and hypertension (Zhao et al., 2020). These complications are particularly common during the fibrosis stage (Targher et al., 2020), with cardiovascular disease identified as the primary cause of mortality among NAFLD patients (Younossi, 2019; Polyzos et al., 2021).

A modeling study by Chris Estes et al., using epidemiological data, has predicted that the prevalence of NAFLD will rise by 21%, reaching 100.9 million cases by 2030. Additionally, decompensated cirrhosis and hepatocellular carcinoma are expected to increase by 137% and 178%, respectively (Estes et al., 2018). As a result, NAFLD is posing an exponentially growing socioeconomic burden.

The liver is composed of two types of macrophages: liver resident macrophages and monocyte-derived macrophages (MoMFs). Both types of macrophages play a significant role in NAFLD. When there is lipid infiltration in the liver, it triggers liver injury and activates Kupffer cells (KCs). KCs then release a large number of inflammatory factors and chemokines, which lead to the recruitment and infiltration of MoMFs (Tacke, 2017); (Roohani and Tacke, 2021). This increase in macrophages causes inflammation and initiates the fibrotic program. During disease progression, KCs are gradually replaced by MoMFs, which involve complex microenvironmental changes and mechanisms (Remmerie et al., 2020).

With the growing prevalence and severity of NAFLD, it is crucial to have a comprehensive understanding of its pathogenesis and to develop and implement therapies that specifically target different aspects of the condition.

## 2 Basic role of KCs

KCs are a significant component of liver sinusoids and represent the largest population of resident macrophages in the human body. They originate from red bone marrow progenitor cells of the yolk sac (Chen et al., 2020a) and can be activated into M1 and M2 states, depending on various signals and inflammatory factors (Li et al., 2017). While M1 macrophages contribute to hepatic steatosis, inflammatory cell recruitment, and fibrosis activation, M2 macrophages have anti-inflammatory and reparative functions. As NAFLD progresses, there is a gradual increase in the M1/M2 ratio and production of pro-inflammatory factors (Kitade et al., 2017) (Luo et al., 2017). This process is dynamic and complex (Alharthi et al., 2020). Apart from their common macrophage functions, such as phagocytosis of exogenous substances, antigen presentation, and immune response, KCs are also involved in several metabolism-related activities, such as iron clearance (Bennett et al., 2020; Wen et al., 2021).

As the liver’s first line of defense, KCs are capable of detecting and intervening in metabolic disorders during the initiation and progression of NAFLD (Cai et al., 2019). Additionally, KCs mediate the initial inflammatory reaction in various types of liver injury (Papachristoforou and Ramachandran, 2022). Throughout this process, KCs interact with various cells, including hepatic sinusoid endothelial cells, hepatic stellate cells (HSCs), neutrophils, monocytes, T cells, dendritic cells, and others, through crosstalk (Matsuda and Seki, 2020; Shan and Ju, 2020; Carranza-Trejo et al., 2021). The malfunction of KCs can lead to and worsen several liver diseases, including NASH, viral hepatitis, alcoholic cirrhosis and fibrosis, and cholestasis (Sato et al., 2016; Li et al., 2020a; Tran et al., 2020; Elchaninov et al., 2022). Thus, KCs are closely associated with the occurrence and development of NASH and the formation of a series of pathophysiological reactions.

**FIGURE 1:**
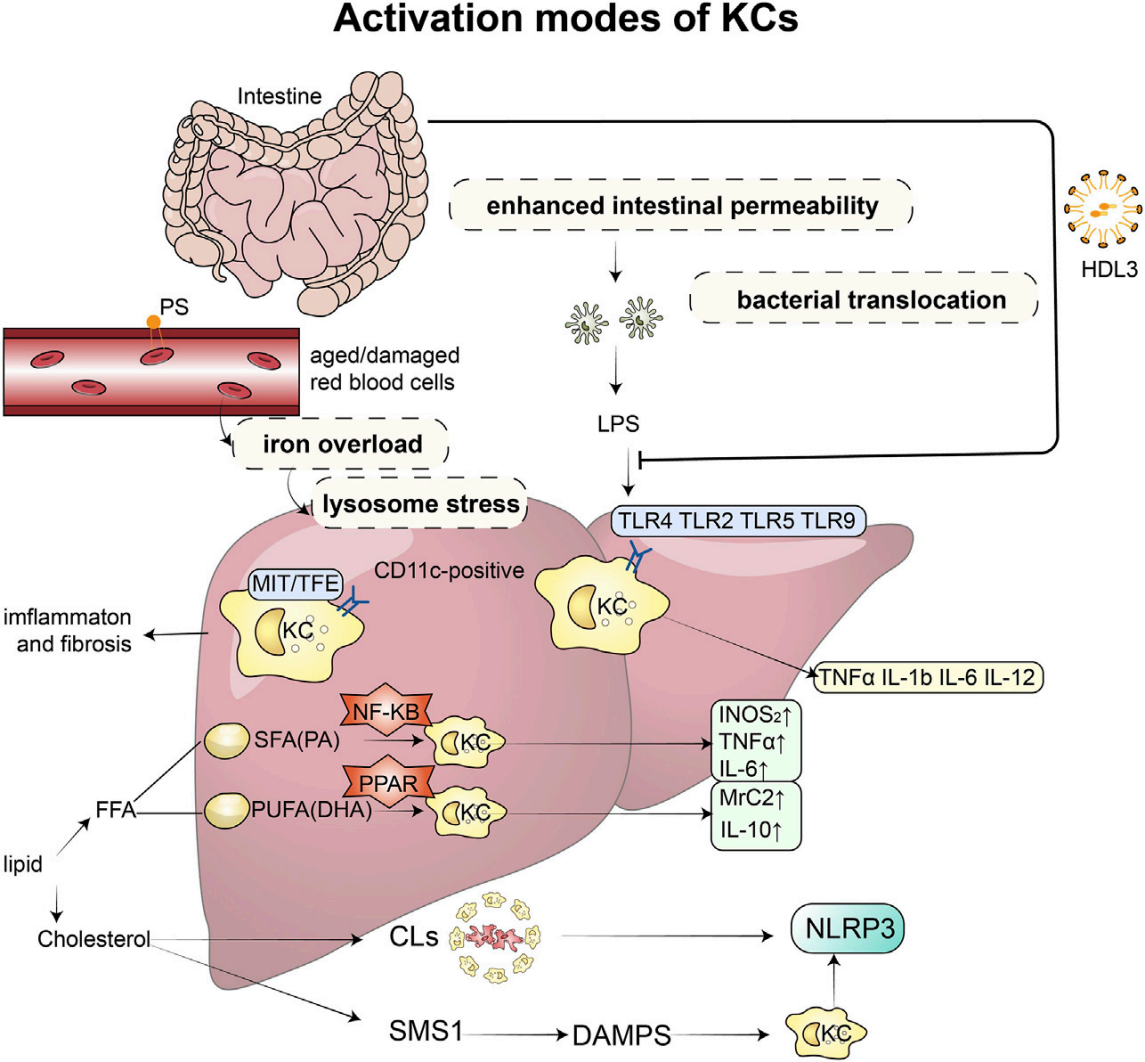
Activation modes of KCs and related mechanisms: Excessive accumulation of lipids can disrupt the microbial balance in the intestine, impairing the function of the mucosal barrier and increasing permeability, which in turn leads to the release of endotoxins. These endotoxins then bind to toll-like receptors (TLR2, TLR4, TLR5, and TLR9) on KCs, triggering the production of inflammatory factors, such as TNF-α, IL-1β, IL-6, and IL-12. However, this binding process can be prevented by HDL3. KCs also play a role in recognizing and taking up senescent erythrocytes, which can cause iron overload and lysosomal stress, leading to intrahepatic inflammation and fibrosis. When stimulated by saturated and unsaturated fatty acids, KCs produce different inflammatory factors and M1 and M2 markers through the PPAR-γ and NF-κB signaling pathways, respectively. Increased and activated KCs surround dead hepatocytes with fatty degeneration, forming typical crown-like structures that contain a large amount of cholesterol crystals and release NLRP3 inflammatory factors. Furthermore, free cholesterol induces the expression of sphingomyelin synthesis (SMS1), which mediates diet-induced hepatocyte pyroptosis. The release of damage-associated molecular patterns (DAMPs) from hepatocytes undergoing pyroptosis can activate NLRP3 inflammasomes in KCs. Abbreviations: KCs, Kupffer cells; TLR, toll-like receptors; LPS, lipopolysaccharide; TNF, Tumour Necrosis Factor; IL, Interleukin; HDL, high-density lipoprotein; SMS1, sphingomyelin synthesis; DAMPs, damage-associated molecular patterns; FFA, free fatty acid; SFA, saturated fatty acids; PA, palmitic acid; PUFA, n-3 polyunsaturated fatty acids; DHA, docosahexaenoic acid; CLs, crown-like structures; NLRP3, NOD-like receptor thermal protein domain associated protein 3.

In the present work, we discussed the links between KCs and the occurrence and development of NASH, focusing on the different activation pathways of KCs and recent research in these areas. Figure 1 illustrates activation modes of KCs and related mechanisms briefly.

## 3 Activation of KCs

### 3.1 Intestine-liver axis

The intestinal-liver axis represents the connection and interaction between the liver and the gastrointestinal tract in physiological processes. Specifically, the liver produces bile acids, IgA, and antimicrobial peptides that are transported to the intestinal tract through the biliary tract, while the intestinal tract carries metabolites to the liver through the portal vein (Tripathi et al., 2018). These signals are responsible for maintaining the physiological immune cell group in the intestine and normal metabolic reactions, which are well-tolerated by the liver. However, in patients with NASH, excessive lipid accumulation can cause a microbial imbalance that impairs the function of the intestinal mucosal barrier, increases permeability, and releases endotoxins (van der Heide et al., 2019). Consequently, bacteria and their products enter the bloodstream, leading to an increased concentration of lipopolysaccharide (LPS) in the liver (Bruneau et al., 2021).

In patients with NAFLD and NASH, there is commonly an increased level of *Bacteroides* and colonization of *Proteobacteria*, *Enterobacteriaceae*, and *Escherichia coli,* while there is a decreased level of *Puccinia* and *Corynebacterium faecalis* (Wu and Tian, 2017). A 2018 study has compared the intestinal microbiota in the feces of healthy individuals and NASH patients, finding that the hematopoietic cell marker differentiation cluster CD45^+^ and the KC marker CD163+ are higher in NAFLD (Schwenger et al., 2018). This indicates a potential relationship between the intestinal microbiota and KC activation. Moreover, the destruction of the intestinal barrier can activate specific types of KCs and exacerbate the activation of RIP3 signaling pathways in liver tissue, leading to a series of cascade reactions (Zhang et al., 2021a). As a result, the intestinal microbiota has become a non-invasive biomarker for diagnosing and evaluating NAFLD (Fianchi et al., 2021). Antibiotic treatment and stool transplantation are emerging as new strategies for preventing and treating NAFLD (Fang et al., 2022). Modifying the microbiota has been identified as a potential therapeutic approach for NAFLD by modulating the activation of KCs. Several studies have shown that specific strains of probiotics, such as *Lactobacillus paracasei* (Sohn et al., 2015) and *Lactobacillus pentosus strain* S-PT84 (Sakai et al., 2020), can mitigate hepatic steatosis by inducing the polarization of KCs and macrophages towards the M2-like phenotype in the NASH model.

The role of LPS from the surface of Gram-negative bacteria in metabolic liver disease has been widely studied. Once intestinal LPS reaches the liver, activated KCs express various inflammatory cytokines, such as NLRP3, TNF-α, IL-1β, IL-6, IL-12, IL-10, IL-18, and others, contributing to the progression of NASH (Yu et al., 2017; Elchaninov et al., 2019; Ferro et al., 2020). Recent studies have shown that the LPS-Toll-like receptor (TLR) signaling pathway plays a key role in the communication between the intestinal tract and liver in NASH. TLR2, TLR4, TLR5, and TLR9 are all related to the pathogenesis of NAFLD (Miura and Ohnishi, 2014). The number of TLR4 + macrophages is significantly increased in NASH patients, and the increase in TLR4 expression is offset by KC consumption (Kazankov et al., 2019), suggesting that active TLR4 expression may be largely contributed by KCs.

In a study conducted in 2020, transgenic mice fed a high-fat diet (HFD) are used as the model, and LPS is found to activate Yes-related protein (YAP) located in KCs through AP-1 transcription in macrophages/KCs, up-regulating pro-inflammatory cytokines, such as MCP-1, TNF-α, and IL-6. This effect is blocked in TLR4-depleted macrophages (Song et al., 2020), suggesting that this link is TLR4-dependent. The activation of the LPS-TLR signal also triggers a specific defense mechanism. The gut produces a unique high-density lipoprotein (HDL) subspecies called HDL3, which, upon release into the portal blood, can combine with LPS and LPS-binding protein (LBP) to form a complex. This prevents LPS from binding with TLR4 in KCs and effectively suppresses the inflammatory response of KCs (Han et al., 2021a). Depletion of LBP in a mouse model has been shown to reduce the occurrence of diet-induced NAFLD to some extent, and its effect is closely related to TLR4 (Jin et al., 2017), suggesting that targeting LBP may be beneficial for human NAFLD. Some researchers have proposed the concept of the Gut-Pancreas-Liver Axis to enhance the understanding of the relevant mechanism (Svegliati-Baroni et al., 2020). Over the past 5 years, researchers have conducted studies on therapeutic drugs and targets for the LPS-TLR signaling pathway, which are summarized in Table 1.

**TABLE 1:** Research on therapeutic drugs and targets for LPS-TLR signals in past 5 years. Abbreviations: HMGB1, High Mobility Group Box 1; TLR, Toll-like receptor; TNF, Tumour Necrosis Factor; IL,Interleukin; OMT,Oxymatrine; Nox4, NADPH oxidase 4; PDK, Pyruvate dehydrogenase kinase; TIR,Toll/interleukin-1 receptor; TCF, total cellular fluid; LAB, lactic acid bacteria; UDCA-18: 1LPE, ursodeoxycholyl oleoyl-lysophophatidylethanolamide; RIP3, receptor-interacting kinase-3.

Gene/protein/Compound/Drug	Animals/cells	Model/treatment	Mechanism/signaling pathway	Influence to pro-inflammatory cytokines	Influence to LPS-TLR signal	Reference
UTI combined with TM	rats	liver and kidney injury mediated by LPS	downregulate the expression of High Mobility Group Box 1 (HMGB1), Toll-like receptor (TLR) 4 and Nuclear Factor (NF)-κB phosphorylation	reduce secretion of IL-6, IL-1β, TNF-α and NO	(−)	Zhang et al. (2022a)
Oxymatrine (OMT)	rats	acute liver failure (ALF) induced by lipopolysaccharide (LPS)/D-galactosamine (D-GalN)	Inhibit hepatocyte apoptosis through the TLR4/PI3K/Akt/GSK-3β signaling pathway	downregulate expression of TNF-α and IL-1β	(−)	Zhang et al. (2017)
NADPH oxidase 4(Nox4)	mice and Huh7 human hepatoma cells	Chronic liver infammation model induced by LPS	mediate LPS-TLR4 signaling in hepatocytes via NF-ĸB and AP-1 pathways	LPS induced TNF-α levels were subsided in the primary hepatocytes of Nox4 knockout mice	(+)	Singh et al. (2017)
Scoparone	mice	NAFLD model induced by methionine and choline-deficient (MCD) diet	Alleviate NASH- and lipopolysaccharide (LPS)-induced immune responses in macrophagespartly by blocking TLR-4/NF-κB signaling in a dose-dependent manner	reduced secretion of MCP-1, TNF-α, iNOS,F4/80, IL-1β and IL-6	(−)	Liu et al. (2019)
Pyruvate dehydrogenase kinase (PDK) 2	mice	endotoxin shock model mediated by LPS	enhance TLR4-MAPK pathway activation and modulate TLR4-mitogen-activated protein kinase signaling and inducing the production of proinflammatory cytokines in macrophages and DCs	increase secretion of IL-6, IL-12, and TNF-α	(+)	Li et al. (2022)
Toll/interleukin-1 receptor (TIR)1	mice	sepsis model induced by LPS	block TLR4 pathway activation via both MyD88-dependent and -independent (TRAM- and TRIF-dependent) pathways	suppress secretion of NO,ROS,TNF-α, IL-12p40, and IL-6	(−)	Kwon et al. (2019)
total cellular fluid (TCF) of lactic acid bacteria (LAB)	Human hepatoma HepG2 cells and THP-1 human monocytic cell line	hepatic steatosis (HS) model	diminish levels of the expression of cytokines via modulation of the expression of TLR-negative regulators, MAPK and NF-κB pathways	reduce secretion of IL-6、IL-8、MCP-1和TNF-α	(−)	Kanmani et al. (2019)
bile acid-phospholipid conjugate ursodeoxycholyl oleoyl-lysophophatidylethanolamide (UDCA-18:1LPE)	THP-1 human monocytic cell line	macrophage phenotype induced by LPS	inhibit LPS-induced recruitment of adaptor proteins into lipid rafts leading to the suppression of p38, MKK4/MKK7, JNK1/2, and NF-κB signaling	reduce secretion of TNF-α, IL-1β and IL-6	(−)	Horvatova et al. (2018)
apilarnil	rats	liver injury induced by LPS	prevent LPS-induced liver damage by inhibiting the TLR4/HMGB-1/NF-κB signaling pathway	downregulate expression of TLR4, HMGB-1, NF-κB, iNOS, TNF-α, IL-1β, IL-6	(−)	Doğanyiğit et al. (2020)
receptor-interacting kinase-3 (RIP3)	mice	NAFLD model induced by HFD	RIP3 knockdown inhibit the expression of lipogenesis-associated genes, inactivate NF-κB pathway and NLRP3 inflammasome, RIP3-mediated effects are dependent on its interaction with TLR-4 and Nrf-2/HO-1	TNF-α,IL-6, IL-1β, IL-18 and CCL-2 is eliminated by the loss of TLR-4	(+)	Chenxu et al. (2019)
Baicalin	chickens	liver inflammation in chicken induced by LPS	negatively regulate of inflammatory mediators through the downregulation of TLR4 expression and the inhibition of NF-kB activation	dose-dependently suppress the production of IL-1β, IL-6 and TNF-α	(−)	Cheng et al. (2017)
*Lactobacillus* plantarum Lp2	mice	liver injury induced by LPS	activate the signal pathway of TLR4/MAPK/NFκB/NRF2-HO-1/CYP2E1/Caspase-3 and regulate the expression of related proteins	reduce secretion of IL-6 and TNF-α	(−)	Chen et al. (2022)

### 3.2 Iron metabolism

Iron is closely associated with NAFLD, as hypoferritinemia is observed in one-third of patients with NAFLD and metabolic syndrome (MetS) (Rametta et al., 2020). Activation of iron regulatory protein (IRP) has been found to increase the expression of divalent metal transporter 1 (DMT1) in patients with NAFLD, resulting in increased iron absorption in the gastrointestinal tract (Hoki et al., 2015); (Miyanishi et al., 2019). Excess iron subsequently affects disease progression by altering macrophage polarization (Handa et al., 2019), interfering with insulin receptor expression and insulin secretion, and modifying the gut microbiome (Mayneris-Perxachs et al., 2021); (Feng et al., 2022). Maintaining normal iron metabolism is an essential auxiliary function of KCs, and intracellular iron overload is one way to activate them. As red blood cells approach senescence, their aging signals on the ectoplasmic phosphatidylserine (PS) surface are recognized by receptors present on splenic red pulp macrophages (RPMs) and KCs. The most abundant receptors that identify these aging signals are the TAM receptors AXL and MERTK, TIM4, and the Fc receptor CD16 (Slusarczyk and Mleczko-Sanecka, 2021). Located in the hepatic sinuses, KCs play a crucial role in removing aging red blood cells, heme, and hemoglobin and express related genes involved in the uptake, processing, and recycling of iron (Sukhbaatar and Weichhart, 2018; Bennett et al., 2020; Slusarczyk and Mleczko-Sanecka, 2021). It has been confirmed that transcription factors SPI-C and NRF2 regulate the iron metabolism gene module (Scott and Guilliams, 2018). Moreover, there seems to be a direct relationship between the phagocytic activity of KCs and the extent of NAFLD. A study using superparamagnetic iron oxide-enhanced magnetic resonance imaging (SPIO-MRI) to assess the phagocytic function of KCs in animal models of NAFLD has demonstrated a strong correlation between the severity of disease observed on SPIO-MRI and the degree of hepatic steatosis, inflammation, fibrosis, and SPIO particle deposition in liver tissues (Cheong et al., 2015). These results indicate that SPIO-MRI can be a promising clinical tool.

Recently, Yohei Kanamor et al. have identified a unique structure in NAFLD patients known as a “crown-like structure (CLS).” This structure consists of dead liver cells with large fat droplets surrounded by iron-rich, CD11c-positive KCs. Lysosomal stress induced by iron overload is believed to cause the formation of the CLS, which activates MiT/TFE transcription factors in CD11c-positive KCs and ultimately leads to liver inflammation and fibrosis (Kanamori et al., 2021). Moreover, hepatocytes are capable of producing secretions containing tissue-specific proteins and miRNAs (Jiao et al., 2021). In addition, KCs can be activated by iron-rich extrahepatic vesicles (EVs) to act as scavengers, resulting in iron deficiency in hepatocytes and iron overload in stellate cells in the liver affected by NAFLD/NASH. The disruption of iron homeostasis can further worsen chronic liver disease (Gao et al., 2022). Targeting the breakdown of iron homeostasis may provide potential avenues for NAFLD treatment, such as specific therapies for iron, like iron chelation, or blocking the production of iron-containing EVs.

Ferroptosis, a type of regulated cell death (RCD), is believed to play a crucial role in triggering immune cell infiltration and inflammatory responses in NASH (Tsurusaki et al., 2019). A series of experiments have been conducted to explore the impact of ferroptosis on NASH using mice fed methionine-choline deficient (MCD) diets. The results of these experiments reveal that the administration of RSL3, a ferroptosis inducer, exacerbates serum lipoatrophy and inflammatory parameters in the mice (Qi et al., 2020). Conversely, treatment with deferoxamine (DFO), an iron-chelating agent, effectively mitigates these negative effects. The imbalance in iron metabolism that leads to lipid peroxidation and the accumulation of reactive oxygen species (ROS) is a critical characteristic of ferroptosis (Feng et al., 2022); (Wang et al., 2022). Inhibition of lipid ROS associated with ferroptosis has been found to improve the accumulation of free lipid droplets in a hepatocyte culture model (Li et al., 2020b). However, it remains to be verified whether this relationship is directly linked to the activation of KCs. Meanwhile, KCs, as well as other macrophages, can regulate ferroptosis by releasing inflammatory factors. IL-6 released from KCs polarized towards M1 triggers ferroptosis by inducing ROS-dependent lipid peroxidation and disrupting iron homeostasis (Han et al., 2021b). TNF-α secreted by M1 induces acyl-CoA synthetase 3, thereby promoting the production of lipid droplets (Jung et al., 2020), creating the conditions for ferroptosis. ROS produced by KCs can induce ferroptosis through lipid hydroperoxides or by depleting antioxidants, such as GSH or glutathione peroxidase 4 (GPX4), in amino acid metabolism (Yang et al., 2022). However, further experiments are needed to link KCs directly to ferroptosis.

### 3.3 Lipid metabolism

The liver is a crucial organ that plays a vital role in lipid metabolism. It is responsible for various processes, such as lipid digestion, absorption, transportation, catabolism, and anabolism, all of which are closely linked to the liver’s function. In normal human metabolism, the liver processes a significant amount of fatty acids every day and stores only a small fraction of fatty acids as triglycerides (<5%) (Alves-Bezerra and Cohen, 2017). However, this delicate balance in lipid metabolism is disrupted in cases of obesity or excessive nutrient intake, which leads to the accumulation of significant amounts of triglycerides within hepatocytes. This subsequent increase in the influx of fatty acids into the liver triggers a cascade of reactions that ultimately results in the development of organic lesions of NAFLD.

In an early experiment on NASH induced by an HFD, the accumulation of lipid droplets causes a unique pro-inflammatory phenotype of KCs, and it disrupts lipid metabolism at the gene level. As a result, the KCs secret more IFNA1 (interferon 1), TNF, IL-10, CCL2 (C-C-C theme chemokine ligand 2), and CCL5, which leads to inflammation. This pro-inflammatory phenotype can be reversed by inhibiting adipogenesis in these KCs with 5-tetracycloxy-2-furoic acid, which slows down disease progression (Leroux et al., 2012). Additionally, KCs express high levels of the class A scavenger receptor (SR-A), which exhibits a strong affinity for modifying lipoproteins. Intrahepatic steatosis reduces its ability to clear LPS, leading to the progression of NASH (Tirosh, 2018). Therefore, these two mechanisms are intrinsically coupled. Research has shown that the chemical consumption of KCs in the initial stage of NAFLD can reduce the severity of liver injury, emphasizing the crucial role of KCs (Peiseler et al., 2022).

Fatty acid activation in KCs has been found to be closely associated with the NLRP3 inflammasome. In a study that utilizes primary KCs extracted from mouse liver as a model, stimulation with palmitic acid (PA) is shown to promote the formation of the mitochondrial DNA (mtDNA)-NLRP3 inflammasome complex *in vitro*. Depletion of NLRP3 partially inhibits the pro-inflammatory effects of PA (Pan et al., 2018). Adropin, on the other hand, has been shown to directly inhibit the activation of PA-induced NLRP3, reducing the levels of caspase-1 and IL-1β proteins and mRNAs in KCs (Yang et al., 2021a). These findings provide new insights into the study of NLRP3 inflammasomes in NAFLD.

TLR4 on KCs functions as a sensor of free fatty acid content, detecting an excess of fatty acids in the liver and triggering the release of TNF-α (Diehl et al., 2020). However, not all fatty acids have the same effect on KCs. Treatment with saturated fatty acids (SFAs), such as those found in an HFD, strongly induces the expression of iNOS2, TNF-α, and IL-6 in KCs. In contrast, treatment with unsaturated fatty acids, such as docosahexaenoic acid (DHA), primarily induces the expression of M2 markers, including Mrc2 and IL-10, which activate the PPAR-γ and NF-κB signaling pathways (Luo et al., 2017). This finding suggests that disease progression can be modified by altering the polarization direction of KCs. Table 2 summarizes the results of studies investigating therapeutic drugs and targets for the induction of KCs with fatty acid in models of NAFLD and NASH in the past 5 years, the active ingredient involved eventually reduced the inflammation and progression of the disease, drugs targeting fibrosis caused by FFA-activated KCs remain to be investigated.

**TABLE 2:** Results of studies investigating therapeutic drugs and targets for the induction of KCs with fatty acid in models of NAFLD and NASH in the past 5 years abbreviations: IQ, Isoquercetin.

Gene/protein/Compound/Drug	Cells/animals	Model/treatment	Mechanism/signaling pathway/conclusion	Influence to pro-inflammatory cytokines	Reference
NLRP3 inflammasome	Primary mice KCs, NLRP3 knockout (NLRP3−/−)male mice	KCs induced by PA	FFA can act as a mechanistic activator of NLRP3 inflammasome in KCs, triggering the NLRP3 inflammasome activation and IL-1β and IL-18 secretion	increase secretion of IL-1β and IL-18	Cai et al. (2017)
ASK1	Primary mice KCs	KCs induced by PA	Palmitic acid induces ASK1/p38MAPK activation and protects H/R damage of steatotic KCs	—	Imarisio et al. (2017)
toll-like receptor 3 (TLR3)	Primary mice KCs, TLR3 knockout mice	KCs induced by PA	TLR3 knockout mice fed high-fat diet showed severe hepatic inflammation accompanied by nuclear factor-κB and IRF3 activation which is mainly induced by the activation of KCs	—	Lee et al. (2018)
PPAR-γ agonist (rosiglitazone)	Primary mice KCs	KCs induced by PA and DHA	Different dietary fatty acids exert opposite effect on KCs polarization, involving activation of PPAR-γ and NF-κB signaling pathway, PPAR-γ agonist alleviates HF diet-induced M1 Kupffer cell polarization	reduce secretion of IFN-γ, TNF-α, IL-1β and IL-6	Luo et al. (2017)
a7nAChR agonist	Primary mice KCs, a7nAChR knockout (a7KO) chimeric mice	KCs induced by PA and/or LPS	The a7nAChR agonist promoted STAT3 phosphorylation and suppressed p65 phosphorylation under TLR pathway stimulation by LPS	reduce secretion of MCP-1, TNF-α, IL-1β, IL-12and IL-6	Nishio et al. (2017)
mitochondrial DNA	Primary mice KCs, NLRP3 knockout (NLRP3−/−) male mice	KCs induced by PA	PA effectively upregulated the expression of the NLRP3 inflflammasome and induced the formation of mtDNA-NLRP3 inflflammasome complexes	—	Pan et al. (2018)
Isoquercetin (IQ)	Primary mice KCs	KCs induced by PA and LPS, KCs and hepatocytes are co-cultured	IQ can improve NAFLD by the activation of AMPK pathway or suppress TGF-β signaling by TGF-βR1-SMAD2/3	reduce secretion of TGF-β	Qin et al. (2018)
RIP1 kinase	Primary mice KCs, Rip1K45A/K45A mice	KCs induced by LPS and PA or OA	RIP1 kinase activity played a key role in mediating the cell death and inflflammasome activation induced by saturated fatty acid PA in both resident hepatic macrophages and bone marrow-derived macrophages	increase secretion of IL-1β	Tao et al. (2021)
adropin	Primary mice hepatocytes and KCs	KCs and hepatocytes are incubated in the presence or absence of PA or PA plus adropin	Adropin inhibits PA-induced NLRP3 inflflammasome activation in hepatocytes and KCs	reduce secretion of IL-1β	Yang et al. (2021a)
Dual TBK1/IKKε inhibitor (amlexanox)	Primary mice hepatocytes and KCs	KCs and hepatocytes are induced by PA	amlexanox attenuated the severity of PA-induced hepatotoxicity *in vitro* and lipoapoptosis by the inhibition of TBK1/IKKε-NF-κB and/or IRF3 pathway in hepatocytes and KCs	suppress the production of IL-1β, IL-6 and TNF-α	Zhou et al. (2020)
Liraglutide	Primary mice KCs	KCs induced by PA and/or Liraglutide	Liraglutide improves mitochondria function and suppresses the activation of the NLRP3 inflflammasome	reduce secretion of IL-1β and TNF-α	Zhu et al. (2018)

In recent years, there has been extensive research on the role of cholesterol in the development of NASH. It has been found that hepatic free cholesterol overload can lead to lipotoxicity, which in turn causes endoplasmic reticulum stress, mitochondrial dysfunction, and toxic oxygen sterol formation, ultimately resulting in hepatocyte inflammation and fibrosis (Horn et al., 2022). This process is particularly relevant in the context of the multiple-hit pathogenesis model, which suggests that insulin resistance, adipose tissue hormone secretion, intestinal microflora, and genetic and epigenetic factors interact to cause NASH in individuals with genetic susceptibility (Buzzetti et al., 2016). Transcriptomics studies have also shown that cholesterol and fat synergistically alter the phenotype of macrophages (McGettigan et al., 2019). Furthermore, immunohistochemical (IHC) testing of the liver macrophage marker Iba-1 has demonstrated that the number, size, and staining area of positive macrophages increase with increasing dietary cholesterol (Yoshii et al., 2021).

As mice and humans cannot synthesize cholesterol *de novo*, KCs can only obtain cholesterol by taking up residual lipid droplets from dead steatotic hepatocytes or by binding to scavenger receptors (SRs) containing oxidized low-density lipoprotein (ox-LDL) particles (Ioannou et al., 2017; Horn et al., 2022). However, disordered uptake of ox-LDL by SRs can lead to the accumulation of lysosomal cholesterol and trigger inflammatory responses in KCs (Ioannou, 2016). In contrast to the “crown-like” structures mentioned earlier, typical crown-like structures are formed by increased and activated KCs surrounding dead hepatocytes with fatty degeneration that contain a large amount of cholesterol crystals. This distinct feature makes NASH significantly different from ordinary liver steatosis (Ioannou et al., 2013). Relevant experiments have shown that this process is related to the activation of NLRP3 inflammatory bodies in KCs exposed to crown-like structures by cholesterol crystals. Treatment with ezetimibe and statins can dissolve the structure and delay the progression of NASH (Ioannou et al., 2015).

KCs are activated by cholesterol through various mechanisms, including the liver x receptor (LXR) *α* (NR1H3) and LXRβ (NR1H2), which are critical nuclear receptors involved in cholesterol absorption, excretion, metabolism, transcriptional regulation, and cholesterol homeostasis regulation, and are also associated with hypertriglyceridemia and liver steatosis (Wang and Tontonoz, 2018); (Russo-Savage and Schulman, 2021). LXRα is highly expressed in rat KCs, and loss of its function results in cholesterol accumulation in hepatocytes, KC activation, and a reduction in natural killer T (NKT) cells (Endo-Umeda and Makishima, 2019). In an *in vitro* experiment involving KCs and hepatocytes, selective activation of intestinal LXR results in the reversal of cholesterol transport, an increase in the anti-inflammatory effect of HDL cholesterol levels through its interaction with the SRB1 receptor, and the conversion of LPS-stimulated KCs from the M1 phenotype to the M2 phenotype. This ultimately leads to a reduction in TNF-α-induced oxidative stress in hepatocytes (Pierantonelli et al., 2020). These findings suggest that the gut-liver axis represents a potential therapeutic target with cross-interaction properties.

Cholesterol-induced macrophage activation is a critical characteristic of atherosclerosis and portal vein inflammation in NAFLD. A study on surgical specimens of NAFLD has found that free cholesterol and oxLDL co-localize in the portal vein wall, with relatively high levels of IL-1β found in these accumulation areas (Ho et al., 2019). However, uptake of ox-LDL by KCs may partially inactivate lysosomal enzymes, inhibiting the efflux of free cholesterol from lysosomes. Accumulation of trapped cholesterol can lead to liver inflammation (Bieghs et al., 2013; Ioannou, 2016), ultimately increasing the incidence of NASH.

Hepatocyte pyroptosis, an inflammatory cell death resulting from caspase 1-mediated activation of inflammatory corpuscles, such as NLRP3 and AIM2 (Shojaie et al., 2020); (de Carvalho Ribeiro and Szabo, 2022), has a complex relationship with NASH. Free cholesterol induces the expression of sphingomyelin synthesis (SMS1), which mediates diet-induced hepatocyte pyroptosis. DAMPs released by hepatocytes undergoing pyroptosis increase IL-1β in an SMS1-dependent manner, activating NLRP3 inflammasomes in KCs (Koh et al., 2021). Thus, inhibiting SMS1 may become a potential therapeutic or preventive target.

Additionally, Shi-You Jiang et al. have discovered an SREBP pathway inhibitor, 25-hydroxycholesterol (25-HC), which does not activate the liver x receptor. 25-HC can significantly reduce the ratio of cholesterol crystal to crown-like structure, improve insulin sensitivity (Jiang et al., 2022), and prevent the occurrence of NASH to a certain extent.

### 3.4 Reactive oxygen species

ROS, including superoxide anion free radicals (O^2−^) and hydrogen peroxide (H_2_O_2_), are constantly produced as by-products of various liver cell energy metabolism. These species play distinct roles at low and high concentrations (Chen et al., 2020b). However, uncoordinated ROS production and anti-oxygenation defense mechanisms can lead to oxidative stress and tissue damage (Hong et al., 2021), which is closely associated with mitochondrial dysfunction (Karkucinska-Wieckowska et al., 2022). During hepatic steatosis development into NASH, there is an excessive generation of ROS, which triggers lipid peroxidation, mtDNA damage, and cytokine release. These events promote hepatocyte damage and fibrosis (Mansouri et al., 2018); (Yang et al., 2019). A study has shown that nitric oxide, a type of ROS, is produced significantly more in NASH patients than in NAFLD patients. This observation indicates that nitric oxide may serve as a potential inflammatory marker for inflammation progression in NASH (Karkucinska-Wieckowska et al., 2022). Another study by Jin-Mei Yao et al. has focused on retinol-binding protein 4 (rRBP4), revealing that rRBP4 treatment increases intracellular ROS levels and promotes the expression of M1-like macrophage markers, such as iNOS, TNF-α, and IL-1β, while reducing the expression of M2-like macrophage markers, such as IL-10, Arg-1, and YM1. This induces KC polarization to the M1 type, and the process is closely related to the NOX2/NF-κB pathway (Yao et al., 2023).

In the late stage of liver fibrosis in NASH, S100A8, a DAMP considered an inflammatory trigger, can activate the TLR4/NF-κB signaling pathway, leading to upregulation of its target genes NLRP3, IL-1β, and IL-18. This process induces the production of ROS and activates NLRP3 inflammasomes in KCs, ultimately leading to the pyroptosis of macrophages (Liu et al., 2022). However, the use of an esterase-responsive carbon quantum dot dexamethasone (CD Dex) can effectively eliminate ROS in the liver by inhibiting the activation of KCs. This treatment significantly reduces liver injury and collagen deposition, thereby preventing the progression of liver fibrosis (Xu et al., 2022). KCs have a greater impact on ROS-mediated mechanisms than hepatocytes, primarily due to the decreased expression of uncoupling protein 2 (UCP2) in HFD-fed mice (Ma et al., 2021).

KCs and ROS have an interactive relationship, and KCs are known to release bioactive mediators, such as ROS, to surrounding hepatocytes and stellate cells through the activation of NOX2 and TLR signaling pathways (Liang et al., 2016); (Luangmonkong et al., 2018; Maeda et al., 2022). In NASH patients, the accumulation of fat and increased levels of LPS polarize KCs towards the M1 phenotype and result in increased production of ROS and cytokines (Ma et al., 2021). In an *in vitro* human liver model of NASH by coculturing human hepatocytes, umbilical vein endothelial cells (HUVECs), and KCs, activation of primary human KCs with a steatosis-conditioned medium leads to the production of ROS and pro-inflammatory factors (Suurmond et al., 2019).

Numerous studies have demonstrated the efficacy of antioxidant and analog treatments aimed at reducing ROS. For instance, gondoic acid (GA) is observed to effectively downregulate the expression of both COX2 and iNOS antioxidant genes while simultaneously reducing ROS release in LPS-induced KCs (Fan et al., 2022). Furthermore, it has been reported that the classical antioxidant polydatin can enhance miR-200a expression, impede ROS-mediated TXNIP signaling, and prevent NLRP3 inflammasome activation, thereby attenuating fructose-induced lipid deposition and associated inflammatory responses (Zhao et al., 2018); (Tang et al., 2022).

### 3.5 Stimulator of interferon genes (STING)

STING is a pathogen recognition receptor (PRR) located in the endoplasmic reticulum (Couillin and Riteau, 2021). This evolutionarily conserved transmembrane protein (Hopfner and Hornung, 2020) has been shown to contribute to the development of inflammation, connective tissue proliferation, and metabolic disorders in the liver by activating KCs and HSCs (Chen et al., 2021a; Chen et al., 2021b). In a study using liver slices from different cases, researchers have observed that patients with mild or advanced fibrosis in NASH have a higher number of STING+/p-TBK1+ cells than the healthy control group (Wang et al., 2020). This finding suggests that the STING TBK1 signaling pathway is activated in KCs and macrophages during the development of NASH. Yongsheng Yu and colleagues have discovered that in HFD-fed mice, mtDNA in hepatocytes induces the expression of TNF-α and IL-6 in KCs. This effect is attenuated after pretreatment with STING-deficient mice or BAY11-7082, an NF-κB inhibitor. These findings suggest that STING acts as a downstream sensor of mtDNA in NASH patients, leading to NF-κB activation in KCs and the release of inflammatory factors (Yu et al., 2019).

### 3.6 Additional modes of KC activation

Interferon regulatory factors (IRFs) are a family of nine distinct proteins found in mammals that play critical roles in regulating the expression of IFNs and modulating both innate and adaptive immune responses (Negishi et al., 2018). A growing body of literature shows that IRFs can also influence the phenotype and polarization of both KCs and macrophages (Chistiakov et al., 2018). For example, IFN-γ can induce the expression of IRF1 in macrophages through the NF-κB pathway, leading to the production of IL-12, iNOS, and IFN-β, and polarization towards the M1 phenotype (Zhang et al., 2022b); (Zhang et al., 2021b). Conversely, IRF4 has been shown to be involved in M2 polarization via the mTORC2 signaling pathway (Huang et al., 2016).

The interaction between KCs and other cells during the progression of NAFLD is a complex process. Lipotoxic hepatocytes release extracellular vesicles (EVs) containing CXC-chemokine ligand 10 (CXCL10), which contribute to the recruitment and pro-inflammatory activation of macrophages. This mechanism is responsible for exacerbating inflammation during NAFLD progression (Ibrahim et al., 2016; Tomita et al., 2016).

Hepatic blood sinusoidal endothelial cells (LSECs) are among the first cell populations to come into contact with portal blood and liver material (Hammoutene and Rautou, 2019). These cells play a crucial role in regulating KC activation via the NO/cGMP/VASP pathway, exhibiting anti-inflammatory features (Tateya et al., 2011). Dysfunction of LSECs affects the quiescent state of KCs, which is consistent with relevant studies demonstrating that LSEC dysfunction precedes liver inflammation and fibrosis (Peters et al., 2018).

However, under conditions of NASH, LSECs can undergo capillarization (Li et al., 2020c), releasing inflammatory mediators that activate adjacent KCs and exacerbate inflammatory infiltration. This mechanism also plays a critical role in adhesion molecule-mediated cell transport and migration, further contributing to inflammatory infiltration (Miyachi et al., 2017); (Roh and Seki, 2018).

## 4 Conclusion and prospects

The concepts of NAFLD and its recently proposed alternative, MAFLD, are becoming increasingly popular due to a growing emphasis on health. Through various animal models and clinical specimens, researchers have confirmed the crucial role of macrophages in NAFLD. The mechanisms involved in the activation of liver-resident macrophages, known as KCs, are complex and include the intestine-liver axis, iron metabolism, lipid metabolism, ROS, and STING. Given that copper and lipids are independently associated with dyslipidemic disease pathogenesis (Blades et al., 2021), and excess serum copper has been significantly associated with the risk of NAFLD (Chen et al., 2021c; Lan et al., 2021), along with KC counteraction of copper-fructose-induced hepatic steatosis (Song et al., 2018), further *in vivo* and *in vitro* experiments related to copper metabolism should be conducted to investigate the influence of copper metabolism on the role of KCs in NAFLD.

The LPS-TLR signaling pathway is a crucial step in the initiation of KCs and a valuable target for the treatment of NAFLD. Preclinical studies have demonstrated the efficacy of herbal extracts, such as Baicalin and Scoparone. In addition, astaxanthin has been shown to increase M2 macrophages with CD206 and IL-10, while decreasing M1 macrophages with CD11c and CCR2 in a mouse model of NASH, thereby reducing intrahepatic oxidative stress and fibrosis (Ni et al., 2015); (Yang et al., 2021b). Extensive clinical trials and translational studies of these drugs will be necessary in the future. KCs possess a high clearance capacity and are an ideal target for drug delivery. Among the clinically relevant drug delivery systems, hard-shell microbubbles (2 µm), liposomes (100 nm), and polymers (10 nm) have been shown to effectively target KCs (Ergen et al., 2017). Additionally, polymeric nanoparticles (NPs) have been found to be readily recognized and phagocytosed by macrophages after intravenous injection (Kumar et al., 2021). The use of novel drug carrier materials may enable specific drugs (such as gene-silencing siRNAs, signaling pathway blockers, antioxidants, inflammatory factor inhibitors, and others) to be delivered to designated sites within the liver. While numerous drugs have shown promise in reducing inflammation and related complications in NASH by regulating KC activation (Xin et al., 2021); (Yin et al., 2021), there is still a significant gap between animal experiments and clinical trials. Moreover, the heterogeneity and diverse subpopulations of human KCs have further impeded the clinical drug development process. Additionally, the multiple-hit hypothesis requires further experiments to explain the complex mechanisms and crosstalk between abnormal lipid metabolism, mitochondrial oxidative damage, and endoplasmic reticulum stress, thereby improving our understanding of the activation pathways of KCs. By further investigating the activation pathways of KCs, we can reduce the impact of NAFLD on people’s health in terms of onset, development, and prognosis.
